# Detailed insight into the dynamics of the initial phases of de novo RNA-directed DNA methylation in plant cells

**DOI:** 10.1186/s13072-019-0299-0

**Published:** 2019-09-11

**Authors:** Adéla Přibylová, Vojtěch Čermák, Dimitrij Tyč, Lukáš Fischer

**Affiliations:** Department of Experimental Plant Biology, Charles University, Faculty of Science, 128 44 Prague, Czech Republic

**Keywords:** Epigenetics, RdDM, RNA interference, sRNA sequencing, Transcriptional gene silencing

## Abstract

**Background:**

Methylation of cytosines is an evolutionarily conserved epigenetic mark that is essential for the control of chromatin activity in many taxa. It acts mainly repressively, causing transcriptional gene silencing. In plants, de novo DNA methylation is established mainly by RNA-directed DNA-methylation pathway. Even though the protein machinery involved is relatively well-described, the course of the initial phases remains covert.

**Results:**

We show the first detailed description of de novo DNA-methylation dynamics. Since prevalent plant model systems do not provide the possibility to collect homogenously responding material in time series with short intervals, we developed a convenient system based on tobacco BY-2 cell lines with inducible production of siRNAs (from an RNA hairpin) guiding the methylation machinery to the *CaMV 35S* promoter controlling GFP reporter. These lines responded very synchronously, and a high level of promoter-specific siRNAs triggered rapid promoter methylation with the first increase observed already 12 h after the induction. The previous presence of CG methylation in the promoter did not affect the methylation dynamics. The individual cytosine contexts reacted differently. CHH methylation peaked at about 80% in 2 days and then declined, whereas CG and CHG methylation needed more time with CHG reaching practically 100% after 10 days. Spreading of methylation was only minimal outside the target region in accordance with the absence of transitive siRNAs. The low and stable proportion of 24-nt siRNAs suggested that Pol IV was not involved in the initial phases.

**Conclusions:**

Our results show that de novo DNA methylation is a rapid process initiated practically immediately with the appearance of promoter-specific siRNAs and independently of the prior presence of methylcytosines at the target locus. The methylation was precisely targeted, and its dynamics varied depending on the cytosine sequence context. The progressively increasing methylation resulted in a smooth, gradual inhibition of the promoter activity, which was entirely suppressed in 2 days.

## Background

All plant cells need to regulate gene expression in connection with developmental processes and as a reaction to external conditions. Simultaneously, the genetic information must be protected against invasive nucleic acids, mainly transposable elements (TEs). To avoid the detrimental effects of their activity, TEs must be kept inactive. However, TEs are integral components of genomes, frequently interspersed between functional genes, so cells need to differentially regulate the activity of particular regions within a genome [1]. For this purpose, cells possess a wide range of epigenetic tools for labelling chromatin at both the DNA and histone level. Histone labelling is highly complex, including a range of various posttranslational modifications of histone tails and varying representation of histone variants within nucleosomes, whereas DNA is labelled almost exclusively by methylation of cytosines (C) [2, 3].

Chromatin epigenetic marks are generally reversible, but they can also be very stable, especially in plants, where repressive marks are often transgenerationally inherited [4]. Therefore, their establishment has to be well-founded and highly specific. The sequence-specific chromatin repression can be realised either by the DNA-binding domains of transcription factors recruiting the polycomb repressive complex that induce trimethylation of H3K27 (these repressive marks are commonly reset between generations) [5] or by RNA-directed DNA methylation (RdDM). Target recognition in RdDM is based on the complementarity of small RNAs with nascent scaffold transcripts of plant-specific RNA polymerase V (Pol V) [3, 6].

DNA methylation serves as a repressive mark to inactivate gene transcription if it occurs in the promoter region [3, 7]; for TEs, methylation is usually spread along their full length [8]. In plants, DNA methylation is targeted on the C5 position of cytosines and can occur in any C contexts: CG, CHG, and CHH (where H can be A, C, or T). Once established, the methylation marks are maintained in dividing cells in three different ways. First, the methylation of CG is maintained by Methyltransferase 1 (MET1), which methylates C in hemi-methylated CG recognised by protein Variant in methylation 1 (VIM1). This process is tightly associated with DNA replication [9–11]. The other two mechanisms are based on the mutual connection between DNA methylation and histone posttranslational modifications—mainly H3K9me2. The first self-reinforcing loop is responsible for maintaining the methylation in CHG (and in heterochromatin also CHH) contexts by Chromomethylases (CMTs). These enzymes contain chromodomains that specifically bind to H3K9me2, which is likely needed for effective methylation of cytosines in the adjacent DNA [12, 13]. Vice versa, methylated CHG is recognised by SRA domain of histone methyltransferases Kryptonite (KYP, SUVH4) and SUVH5/6, which di-methylate H3K9 in the adjacent nucleosome(s) [14–20]. CHG context is maintained mainly by CMT3 [13, 21, 22]. CMT2 is responsible for methylation of CHH and also partly CHG context in canonical heterochromatin containing histone H1 [13, 22].

The last mechanism of “maintenance methylation” is most important for short TEs and border regions of long retrotransposons [8] and is based on the activity of Domains rearranged methyltransferase 2 (DRM2) in the process of canonical RdDM. RdDM is a part of the RNA interference (RNAi) machinery, which inactivates gene expression not only at the transcriptional (TGS) level, but also at the post-transcriptional (PTGS) level [23, 24]. In RdDM, DRM2 methylates C in a context-independent manner at *loci* that are complementary to small RNAs present in the cell [25]. There are several pathways of RdDM that have been described in plants in recent years. The canonical RdDM primarily serves to maintain CHH methylation in already repressed regions. It involves two plant-specific polymerases, Pol IV and Pol V [26–29]. Pol IV is responsible for the production of transcripts which serve as a source of small interfering RNAs (siRNAs) from genomic regions with inactive chromatin lacking histone H1, whereas Pol V assists in recognition of the target regions. Pol IV is attracted to chromatin via its interacting partner Sawadee homeodomain homologue 1 (SHH1), which binds to H3K9me2 and non-methylated H3K4 [30–32]. From *loci* with these chromatin labels, Pol IV creates 30–40-nt-long transcripts, which are replicated by RNA-dependent RNA polymerase 2 (RDR2) producing dsRNA precursors that are processed by Dicer-like 3 (DCL3) into 24-nt siRNA [33–36]. These siRNAs in association with Argonaute proteins AGO3/4/6 base-pair with nascent transcripts of Pol V and guide DRM2 for the methylation of C on the template DNA [6, 37, 38]. Pol V is primarily recruited to *loci* containing methylated cytosines via interaction with inactive SUVH homologs SUVH2/9 [39–41]. This canonical RdDM pathway serves not only to maintain methylation of genomic regions in *cis* but importantly, it should also allow siRNA-mediated “identity-based” recognition and de novo methylation/inactivation of newly inserted copies of TEs in *trans* [42, 43].

Recognition and de novo silencing of completely novel TEs (or transgenes) are likely expression-dependent and can be mediated by several other non-canonical RdDM pathways [43]. In addition to 24-nt siRNAs produced from transcripts of Pol IV in the canonical pathway, Pol II-dependent 24-nt and 21–22-nt siRNAs were also shown to be involved in the pathways, which are considered responsible for the methylation of DNA in *loci* not transcribed by Pol IV [3, 43]. In addition to typical siRNAs, recently discovered DCL-independent sidRNAs have also been suggested to be initial triggers of de novo DNA methylation of epigenetically naive *loci* [44], though a later study challenged this hypothesis [43].

While the molecular mechanisms of RdDM are relatively well-described at present, less is known about its dynamics. Voucheret already in 1994 showed that transcriptional *trans* silencing could start quickly in developing seed, but complete inactivation might require few weeks [45]. After massive leaf infiltration with Agrobacterium, rapid methylation of T-DNA was detectable in the promoter region just 2–3 day post-infiltration and the levels continued to rapidly accumulate over the 1st week and then steadily up to 21 days [46]. In mitotically dividing cells, the maintenance methylation of newly synthesised DNA strands must be quick enough to ensure the replication of the epigenetic information between the two subsequent S-phases of the cell cycle. It is supposed to be exceptionally fast in the case of CG and CHG sequences. For instance, in human embryonic stem cells, the vast majority of the maintenance CG methylation takes place less than 20 min after replication [47]. However, to our knowledge, there is no information available for the dynamics of de novo RdDM initiation phases. Both a quick and slow model could apply; quick TGS would ensure fast, reliable inactivation of invasive DNA. However, it would also make sense to methylate cytosines slowly or with a certain lapse in time from the initial emergence of siRNAs. siRNAs also allow effective protection at the post-transcriptional level, so the postponed, non-impetuous decision to inheritably inactivate some genomic region by DNA methylation might be advantageous, because it could help to avoid potentially detrimental effects connected with unwanted permanent inactivation. Moreover, Pol V, which is regarded as an indispensable component of all RdDM pathways [3], was shown to be specifically attracted to methylated DNA [40], so the speed of the initial methylation of epigenetically naive *loci* could be restricted. Our results, based on the inducible activation of siRNA synthesis, show that RdDM could be initiated several hours after the appearance of high siRNA levels and that the targeted genomic region could reach practically full methylation in as early as 2 days in mitotically dividing tobacco BY-2 cells.

## Results

**Fig. 1:**
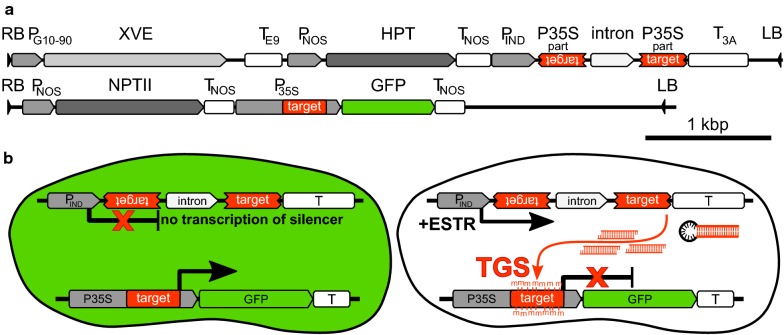
Scheme of the model system. **a** Scheme of the inductor and the target *T*-*DNAs*; **b** expected activity of the T-DNAs in BY-2 cells untreated and treated with β-estradiol; P35S–CaMV constitutive promoter; XVE—a chimeric transcription activator; Pind—promoter activated by estradiol bound to XVE; HPT and NPTII, hygromycin, and kanamycin-resistance genes, respectively (for more detailed description, please see “Methods” and the original paper introducing the XVE-inducible system [70])

The goal of our study was to describe the precise timing and progression of the transcriptional gene silencing (TGS) in its early stages. For this purpose, we used the BY-2 cell line [48] as a model, which allowed us to monitor the process in a highly synchronised and homogeneous culture, which can be studied at a single-cell level [49]. A selected BY-2 cell line stably expressing *GFP*, driven by *CaMV 35S* promoter (*P35S*) [50], was super-transformed with an estradiol-inducible silencing construct composed of an inverted repeat prepared from a part of *P35S* (*IR*-*P35S*) (Fig. 1a). The *IR*-*P35S* transcript was expected to form an RNA hairpin, which should have been processed to siRNAs targeting *P35S* and inducing TGS of the downstream laying *GFP* (Fig. 1b). For the experiment, we chose three independently transformed lines (8, 19, and 35), which showed a high fluorescence level, homogenous silencing response after *β*-estradiol treatment, and which did not show spontaneous self-silencing of the *GFP* gene. To keep the cells in a physiologically invariable state, cultures were continually kept in the exponential phase of growth. Establishment of TGS was monitored for 10 days of continuous *β*-estradiol treatment. In selected timepoints (3, 6, 12, 24 h and 2, 3, and 10 days), we determined the transcript level of the silencer and the target *GFP* gene, GFP fluorescence, promoter cytosine methylation, and presence of promoter-specific siRNAs in the selected lines.

### Monitoring of GFP silencing at the fluorescence and transcript level

**Fig. 2:**
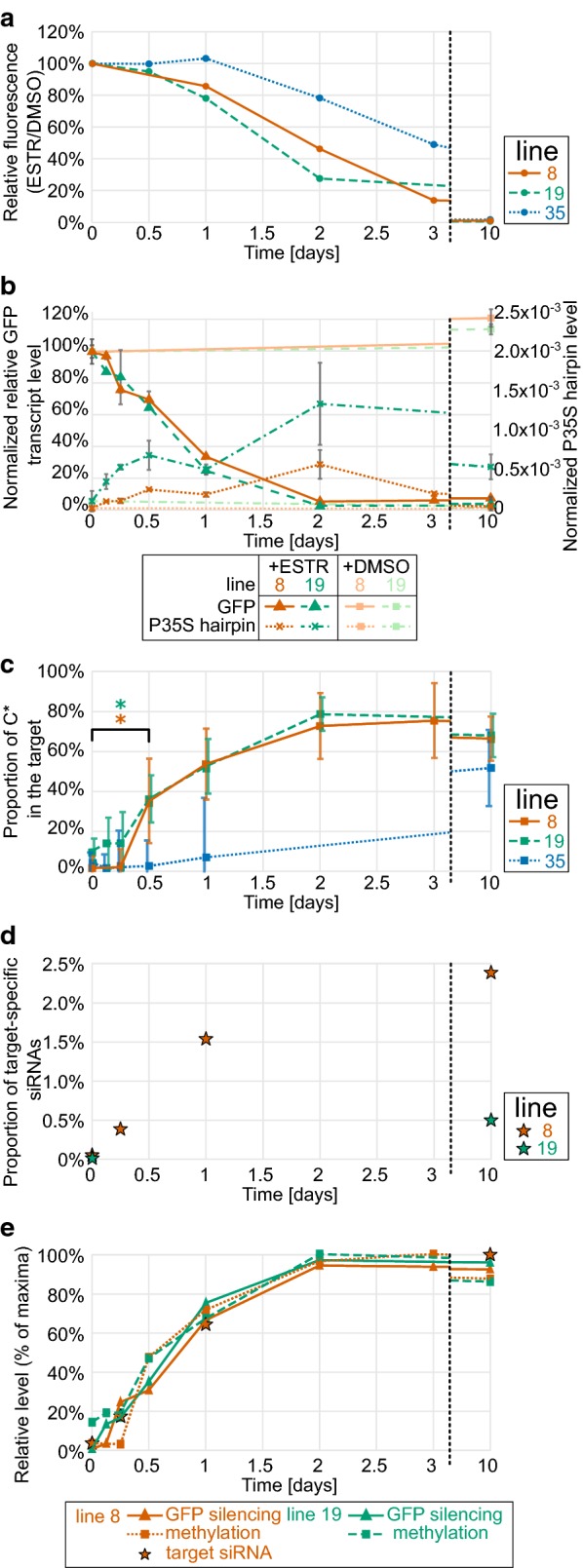
Time course of transcriptional silencing of *P35S::GFP*. β-estradiol was applied at time 0 to three BY-2 lines (8, 19, and 35). The establishment of TGS of *P35S::GFP* was monitored in the selected timepoints at the level of **a** GFP fluorescence (flow cytometry of about 10 thousand cells per sample); **b** transcription of the *GFP* and the *P35S* promoter hairpin (RT qPCR); **c** proportion of methylated cytosines in the target region (means of about 10 clones per sample after bisulfite conversion); **d** proportion of promoter-specific siRNAs (of about 50 mil reads per sample); **e** correlation between the level of *GFP* silencing (i.e., relative decrease in *GFP* expression) and cytosine methylation (relative to the maximal attainable level) and siRNA level (relative to the maximal attainable value; only for line 8). Error bars in (**b**) and (**c**) indicate standard errors, and asterixis in (**c**) indicate a significant difference (Mann–Whitney *U* test, *p* < 0.05) for lines 8 and 19

GFP fluorescence after application of *β*-estradiol was assessed at a single-cell level using flow cytometry of isolated protoplasts. Ten-day *β*-estradiol treatment resulted in a complete loss of detectable GFP fluorescence in all three tested lines (Fig. 2a; Additional file 1). The fluorescence decreased quickly in lines 8 and 19, reaching about 50% of their initial level in 2 days, while the third line 35 responded more slowly (Fig. 2a). The flow-cytometry histograms clearly showed that in lines 8 and 19, the intensity of GFP fluorescence was highly homogeneous in the cell populations during the whole *β*-estradiol treatment (Additional file 1). It indicates that the cells responded synchronously to the induction, allowing a bulk analysis of harvested cells from these lines which provide reliable molecular data representing the progression of TGS in individual cells.

Following the results of the fluorescence analysis, we wondered how transcript levels of the silencer and the target *GFP* changed in the early steps of TGS. For this purpose, we analysed the homogeneously responding lines 8 and 19 using RT qPCR. After the application of *β*-estradiol, the transcript level of *IR*-*P35S* (estimated by amplification of the intron RNA indicating unspliced hairpin level) quickly increased within the first 3 h (Fig. 2b). Transcription of the silencer was followed by a rapid decrease in transcript level of the *GFP* gene in the first 12 h of the treatment (*p* < 0.005 for both lines) by about 30% (Fig. 2b). Afterwards, the transcription gradually declined to less than 5% of the initial transcript level within 2 days. The changes in GFP transcription were highly similar in both tested lines. *IR*-*P35S* transcript levels were, after the initial rise, fluctuating in time and finally decreasing towards the end of the treatment, even though the cells were continually exposed to *β*-estradiol, which might be connected with increased rate of the hairpin RNA cleavage by the action of not only DCLs, but also AGO proteins.

While the synthesis of new GFP protein was almost completely turned off during the first 2 days, the GFP fluorescence decreased by only 50% at the same time. Given that the cultures were exponentially growing, the observed decrease in GFP fluorescence had to result from both GFP degradation and GFP “dilution” in dividing cells. Comparing the speed of the GFP fluorescence decrease and the rate of BY-2 cell divisions (doubling time is about 20 h) [49] clearly indicated that the GFP protein was highly stable in BY-2 cells with a half-life of several days, which caused the observed delay in GFP fluorescence decline.

In summary, the fluorescence and transcription analyses clearly showed that the BY-2 cell populations homogeneously responded to *β*-estradiol and gradually switched off the GFP transcription during the first 2 days of the treatment.

### The onset of *P35S* methylation

We further analysed how the observed decline in GFP expression (onset of TGS) correlated with methylation of the *P35*S. To analyse DNA methylation at a single nucleotide level, we used bisulfite modification of cytosines and subsequent sequencing of about 10 clones per sample. For the amplification, we designed primers, which covered not only the target, but also broader adjacent regions. Within the amplified segment, we obtained information about the methylation state of 90 cytosines in the target (379 nt) and 44 cytosines in the adjacent regions (104- and 82-nt up- and downstreams, respectively). From the 90 cytosines in the target region, there were 13 in CG and 9 in CHG context.

**Fig. 3:**
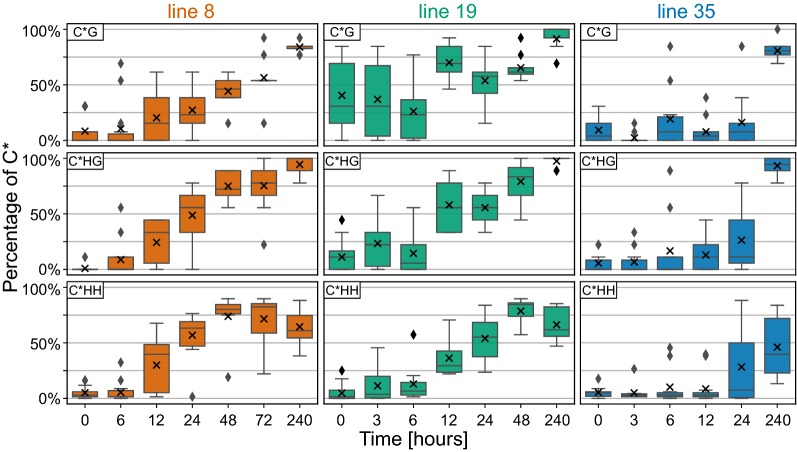
Dynamics of cytosine methylation in different sequence contexts. Changes in cytosine methylation in CG, CHG, and CHH contexts in the target region of *P35S* were determined via bisulfite conversion in three BY-2 lines before and during β-estradiol treatment. The box plots show frequency of methylated cytosines in about 10 sequenced clones per sample. The analysis covered 68 cytosines in CHH, 13 in CG, and 9 in CHG context

The analysis showed that the majority of analysed clones from lines 8 and 35 were practically without methylated cytosines (C*) (Fig. 2c; Additional file 2), and the frequency of non-methylated cytosines matched the experimentally determined efficiency of cytosine conversion, which was about 98% in our experiments. In contrast, in line 19, the median level of initial methylation was as high as about 11%, mainly due to the high proportion of symmetric CG methylation. This methylation might be a remnant of methylation induced by transient expression of the hairpin during the transformation event. Methylation in CHG and notably CHH context was at very low levels in all three lines before the treatment (Fig. 3).

After exposure to *β*-estradiol, the total cytosine methylation in lines 8 and 19 gradually increased and was significant after 12 h (Mann–Whitney *U* test, *p* < 0.05) (Fig. 2c). In the first 2 days, methylation reached its maximum of around 80% of C* in the target region. The methylation status of the target *P35S* region in lines 8 and 19 treated and untreated with *β*-estradiol was further confirmed in selected timepoints by cleavage with methylated DNA specific endonuclease (Additional file 3). The onset of methylation in line 35 was considerably slower, which was consistent with the later decline in GFP fluorescence in this line (Figs. 2a, c, 3). However, on day 10, the methylation reached similar levels in all three lines.

Methylation in CG and CHG contexts gradually increased in time and needed more than 3 days to reach their maximal levels. In contrast, CHH methylation showed a different pattern (Fig. 3). In lines 8 and 19, CHH methylation reached its maximum (median value near 80%) in 2 days and then slightly declined to about 60% until the 10th day. The median values of methylation in CG context on the 10th day of treatment reached 85%, 92%, and 81% in lines 8, 19, and 35, respectively, whereas the methylation of CHG context was practically complete; the median reached 100%, 100%, and 94% in the respective lines.  These results show that the dynamics of methylation establishment differed depending on the cytosine sequence context.

**Fig. 4:**
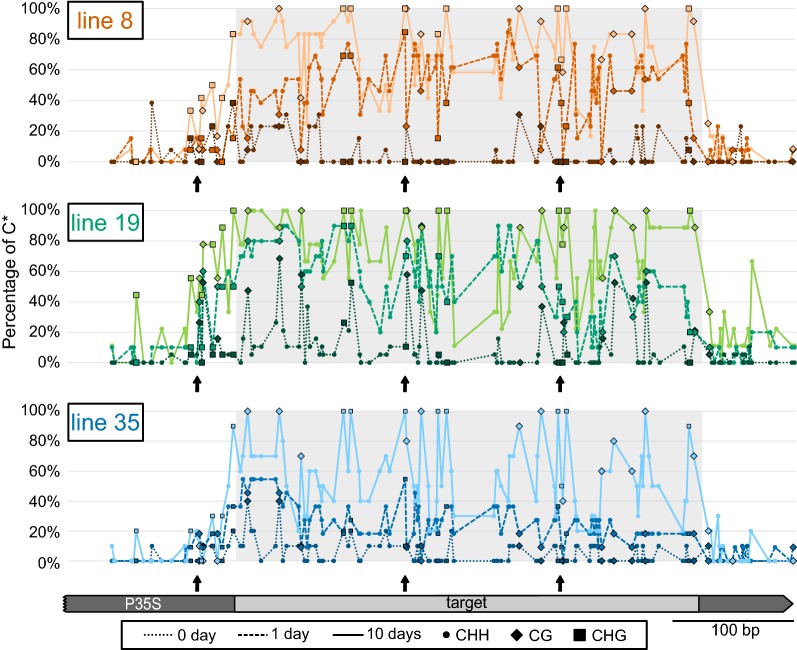
Establishment of cytosine methylation and its distribution along the *P35S* region. The proportion of methylated cytosines on each cytosine position along the target and adjacent regions of *P35S* is shown for the three tested lines before the β-estradiol treatment and after 1 and 10 days of the treatment. Individual cytosine contexts are differentiated: circle for CHH, diamond for CG and square for CHG. Arrows indicate the position of CCG sites

On the 10th day, methylated cytosines were more or less equally distributed along the target region. Even though there were regions and positions with less dense methylation, we could not find any cytosine position, which was utterly resistant to methylation in all analysed clones. The distribution of highly and less methylated cytosines along the target sequence was similar in all three analysed lines (Fig. 4). When focusing on the transient states, it seemed that there was a small preference to initiate methylation in the more upstream part of the target region (Additional file 2). The distribution of C* in incompletely methylated samples from earlier times indicates that the modification of some cytosines was “easier” and they reached their final methylation levels as early as after 24 h of the treatment, whereas other cytosines needed more extended time for the effective establishment of the methylation marks (Fig. 4).

In addition to the *P35S* region targeted by the *IR*-*P35S* construct, our methylation analysis also covered adjacent untargeted regions, which allowed us to evaluate the preciseness of targeting. Cytosine methylation was not absolutely restricted to the target region, but the proportion of methylated cytosines was much lower outside the target region. This external methylation was more prominent in the symmetrical contexts (mainly CHG) (Additional file 2) that was cumulated just upstream of the target region, which might be related to some pre-existing weak methylation present in this region before the treatment (Fig. 4, Additional file 2).

In summary, the induction of the *P35S*-hairpin expression resulted in a gradual increase in *P35S* methylation within the first 2 days of exposure, reaching about 80% of methylated cytosines, which correlated well with the smooth attenuation of the *P35S* promoter activity (Fig. 2e).

### Analysis of small RNAs as a trigger of promoter methylation

Sequence-specific DNA methylation is directed by siRNAs (RdDM) [25], so we performed high-throughput sequencing of siRNAs isolated from lines 8 and 19 at selected timepoints to see the correlation between the presence of promoter-specific siRNAs and promoter methylation. After checking the quality of sequencing output (Additional file 4), all siRNA reads were filtered for only those mapping on any region of the two T-DNAs present in our cells (containing the target *P35S::GFP* and the silencer *XVE::IR*-*P35S* regions; for the silencer, both spliced and unspliced forms were used for the filtering) (Additional files 5, 6: Table S2). The amount and distribution of siRNAs aligned to T-DNAs differed slightly between the two lines. Whereas in line 8, there were practically no siRNAs that aligned outside the target *P35S* region, in line 19 many regions of both T-DNAs were covered with low levels of siRNAs even before treatment (Additional files 5, 6: Table S2). After treatment with *β*-estradiol, siRNAs’ levels increased mainly in the *P35S* target region, but in both tested lines, and more significantly in line 19, some smaller increases were also observed in the hygromycin phosphotransferase (*HPT*) expression cassette (composed of nopaline synthase promoter, *HPT* gene, and nopaline synthase terminator) lying upstream of the *P35S* hairpin (Additional files 5, 6: Table S2).

We further focused on the *IR*-*P35S*, where we analysed which sequences served as a source of siRNAs. As we expected, the alignment of siRNAs on the *IR*-*P35S* showed that the vast majority of siRNAs came from the inverted repeat region. No siRNAs aligned to the intron sequence and the exon/intron or intron/exon interface. Very low levels of siRNAs also originated from the spliced unique loop region of the hairpin, indicating the production of transitive secondary siRNAs (Additional file 5C), though these siRNAs could also originate from some structural rearrangements of the transgene [51]. The relative proportion of these transitive siRNAs did not significantly change during the treatment (Additional file 5C). In the target T-DNA containing full-length *P35S*, we detected no transitive siRNAs which would have expanded a single nucleotide from the target region at least in line 8 (Additional file 5).

**Fig. 5:**
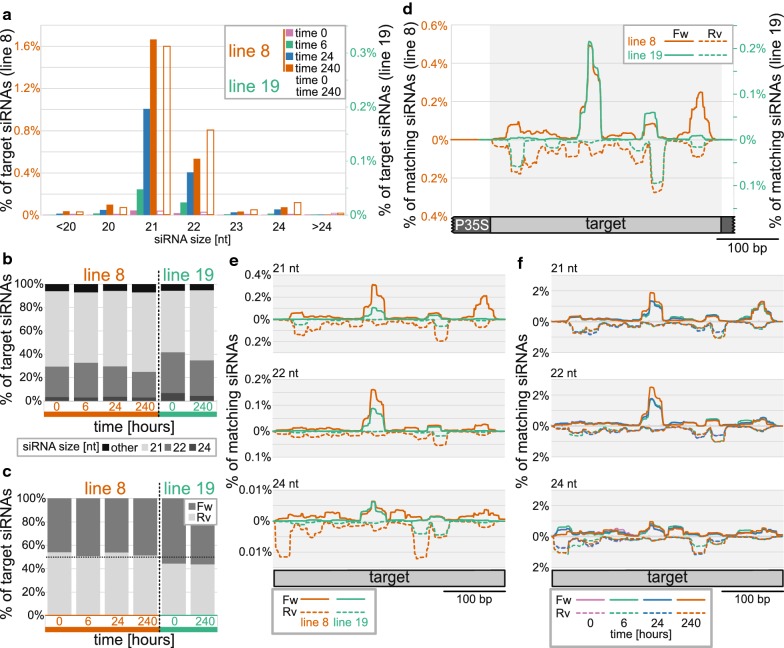
Classification and distribution of siRNA*s* matching with the *P35S* region. All sequenced siRNA*s* were filtered for those matching with the *P35S* target region and further analysed: **a** relative representation of size classes of *P35S* siRNA*s* in all sequenced siRNA*s* (1% corresponds to 1 × 10^4^ siRNAs per million reads; note different scales for the two lines); **b** relative representation of selected siRNA size classes in *P35S*-specific siRNAs; **c** relative representation of forward- and reverse-oriented *P35S*-specific siRNAs; **d** distribution of siRNA*s* isolated from 10-day treated cultures along the promoter sequence depicted as % of all sequenced siRNA*s* (note different scales for the two lines); **e** distribution of selected siRNA size classes along the promoter sequence depicted as % of all sequenced siRNA*s* (siRNA*s* isolated from 10-day treated cultures); **f** time changes in distribution of selected siRNA size classes along the promoter sequence in line 8 (depicted as % of siRNAs of the respective size matching to the target in the respective time)

For subsequent thorough analysis, we used only siRNAs aligning to the target *P35S*. In line 8, the level of these siRNAs gradually increased during the treatment, reaching almost 2.5% of all sequenced siRNAs on day 10. At the same time, in line 19, the level of *P35S* siRNAs was about 5 times lower (Fig. 2d). The length distribution of siRNAs was relatively stable during the treatment, with 21-nt and 22-nt siRNAs being dominant (Fig. 5a). The ratio between 21 and 22-nt siRNAs differed in the two lines and fluctuated slightly during the treatment, but both size classes constantly formed together about 90% of all *P35S* siRNAs in both tested lines. 24-nt siRNAs stayed at an approximately constant level of about 5% of all *P35S* siRNAs during the whole treatment (Fig. 5b).

The siRNAs aligning to the *P35S* were not distributed equally along the sequence, but there were hot- and cold spots with high and low coverage by siRNAs, indicating different stability and/or efficiency in their generation (see the list of the most frequent siRNAs in Additional file 7: Table S3). The distribution of the hot- and cold spots was strongly strand specific. In most positions along the target region, the siRNAs aligned almost exclusively on either the forward or the reverse strand, and rather exceptionally to both of them (Fig. 5d). Despite this, the ratio between forward and reverse siRNAs stayed approximately constant during the treatment at about 1:1 in both tested lines (Fig. 5c). The position of hot- and cold spots was similar for all siRNA size categories. Whereas the distribution of 21- and 22-nt siRNAs was almost identical, 24-nt siRNAs in line 8 aligned to slightly different positions, especially in the most upstream part of the target region (Fig. 5e). The distribution of siRNAs along the sequence was also relatively stable during the whole treatment, though some gradual changes could be seen with increasing duration of the *β*-estradiol treatment; especially in the case of 24-nt siRNAs aligning to the left border of the target region in line 8 (Fig. 5f). Interestingly, low levels of *P35S* siRNAs were already detected in the untreated cells. The size and strand-specific distribution of these sRNAs along the sequence corresponded with the situation observed in the induced cultures, indicating that these siRNAs likely originated from a few sporadically silenced cells, which can also be seen in the flow-cytometry histograms (Additional file 1).

Surprisingly, the distribution of siRNAs along the target region differed between the two analysed lines. Though the position of the main hot- and cold spots was similar, in the case of line 8, the coverage by siRNAs was much more homogeneous. In line 19, there were sharper peaks and also deeper valleys, with only a minimal number of aligning siRNAs (Fig. 5d). The regions with low siRNA coverage in line 19 and higher coverage in line 8 included approx. 30-nt-long border part of the target region. However, there was no obvious difference in the distribution of final methylation along the target region in the two lines on day 10 (Additional file 2, Fig. 4).

In summary, the transcription of *IR*-*P35S* led to the formation of high levels of target-specific siRNAs with the dominant representation of 21- and 22-nt-long classes. The siRNAs aligned unevenly and with strand-specificity along with the target *P35S* region, causing relatively smooth and homogeneous methylation of the whole target region. The levels of target-specific siRNAs gradually increased during the treatment, but there were no dramatic changes in either the representation of siRNA size categories or their distribution along the target region.

## Discussion

Our study describes in detail the dynamics of de novo RdDM in plant cells. Thanks to our highly synchronised BY-2 model system, we could show that induction of DNA methylation and subsequent transcriptional silencing can be a rapid process. Methylation was initiated almost immediately with the appearance of promoter-specific siRNAs, and the first significant difference in methylation was observed as soon as 12 h after induction and caused complete transcriptional silencing within 2 days. Later, however, the methylation pattern developed further. There were some more unexpected features that we described in connection with the establishment of TGS; CG methylation occurring at the target locus prior to the treatment had no effect on the speed of RdDM, the proportion of 24-nt siRNAs was low and stable in the 1st days of DNA methylation, the speed of de novo methylation was much slower than maintenance methylation of newly synthesised DNA strands, and the dynamics of DNA methylation differed depending on the cytosine sequence context. Our study thus opens up new questions that will lead to a better understanding of this biologically important process.

### High levels of specific siRNAs quickly induce methylation

Expression of the *P35S* hairpin led to the gradual accumulation of siRNAs to relatively high levels as compared with the previous reports [52]. *P35S* siRNAs exceeded levels of siRNAs aligning onto well-characterised tobacco transposable elements by a factor of 10 (Tnt1 and Tto1) (Additional file 6: Table S2) [53, 54]. The total levels of *P35S* specific siRNAs differed about five times in the two tested lines, with no effect on the speed of methylation (Fig. 2), indicating that the siRNAs levels were above saturation, so we likely observed the maximal speed of de novo DNA methylation.

The siRNA level sufficient for full, dense methylation in line 19 on day 10 corresponded to the siRNA level observed in line 8 as early as after 6 h of the treatment. However, at that time, only weak methylation was present in line 8 (Fig. 2). It clearly indicates that not only reaching a certain level of siRNAs, but also a certain duration of the exposure to siRNAs was necessary to establish dense methylation of the target region. On the other hand, once established, the dense methylation had to be very quickly introduced to newly synthetized DNA strands after replication, because we observed this dense methylation in the cells that were continually dividing approximately every 20 h [49]. Such faster methylation compared to de novo methylation of a naive locus was likely connected with the presence of the maternal highly methylated DNA strand, since Pol V is effectively recruited to *loci* with methylated cytosines [40].

The onset of dense cytosine methylation was gradual, and the effectiveness of methylation varied slightly depending on the cytosine position within the target region (Fig. 4). However, we did not detect any clear correlation with the position of hot and cold spots of either typical 21–24-nt siRNA (Fig. 5) or longer sRNAs (practically missing in our system) (Additional file 6: Table S2), which could potentially represent sidRNAs that were suggested to participate in the initiation of methylation in epigenetically naive *loci* [44]. The onset of methylation also differed, depending on the cytosine sequence contexts. Methylation in CG and CHG contexts reached higher final levels compared to CHH methylation, which likely reflected the fact that we monitored methylation in dividing cells. CG and CHG methylation could be reintroduced to newly synthesised strands more rapidly and more infallibly after the replication than CHH, because once established, CG and CHG could also be methylated independently of RdDM [18, 55]. Recently, it was shown that external cytosines of CCG sites (a subtype of CHG) in gene bodies could only be methylated when internal cytosines are methylated [56]. Only two such sites present in our target sequence, however, showed the opposite tendency. In line 18 lacking initial CG methylation, the methylation of the external cytosine preceded the methylation of the internal one, which was probably associated with the combined action of DRM2 and maintenance methylation by CMT3. The decrease in CHH methylation observed during prolonged 10-day treatment might result from silencing of the IR construct and a hypothetical decrease in siRNA levels between day 2 (not determined in our siRNA analysis) and day 10. However, the decrease in CHH methylation was equal in both tested lines, though the level of siRNAs on day 10 was 5 times higher in line 8 compared to line 19. Therefore, we prefer an alternative explanation: establishment of high-density CG and CHG methylation might reduce the necessity or efficiency of RdDM, which is then somehow attenuated irrespective the continual presence of high levels of siRNAs.

It should also be noted that the proportion of 24-nt siRNAs was relatively low and stable, as was also reported for IR-derived siRNAs in *Arabidopsis* [52]. This indicates that either the observed levels of 24-nt siRNAs were sufficient to induce effective methylation or that other siRNA sizes participated or were fully responsible for targeting de novo methylation in our system. Involvement of 21-nt and 22-nt siRNAs was clearly demonstrated in the RDR6–AGO6 RdDM pathway [57]. Since there was no relative increase in 24-nt siRNA production during the treatment, it is also unlikely that Pol IV was involved in the generation of siRNAs from this locus, even when it was already repressed and densely methylated on day 10. All detected siRNAs more likely originated from the Pol II hairpin transcript. The high frequency of 22-nt siRNAs indicated the involvement of DCL2, which was shown to stimulate the synthesis of secondary siRNAs through RDR6 activity [58]. Therefore, the detected siRNAs were likely a mixture of primary siRNAs produced from the stem part of the inducer hairpin and secondary siRNAs, presumably originating from the inducer transcript processed by RDR6 after the primary AGO cleavage [59].

This assumption was further supported by the detection of siRNAs originating from outside the dsRNA (stem) region of the hairpin, although levels of these siRNAs were relatively low compared to those originating from the IR region (Additional file 5C). On the other hand, transitivity was completely missing in the target locus. It can be linked to the fact that we targeted a promoter sequence, which is not transcribed or is transcribed only sporadically [60], so the transcripts could not serve as a source of secondary siRNAs. The absence of siRNAs from outside the target region was consistent with practically no CHH methylation outside the target region, showing high preciseness of RdDM targeting, as was suggested from the molecular mechanism of DRM2 action [25]. Such pinpoint targeting is especially important for the inactivation of invasive DNAs (transposable elements) inserted between plant genes, whose expression should not be affected [13].

In contrast to CHH, the methylation of CHG was also relatively high in the adjacent regions and reached practically 100% within the target region, indicating the involvement of CMT3 with less precise targeting, which is based on CMT3 binding to H3K9me2 [12]. This chromatin mark is known to attract not only CMT3, but also Pol IV (via SHH1) [31]. However, our results did not indicate the production of 24-nt siRNAs from *P35S* by Pol IV/RDR2/DCL3 activities, as we see no increase in the proportion of 24-nt siRNA in later times. Therefore, either the Pol IV occupancy was prevented by another chromatin/histone modification (e.g., H3K4me3) [31], or the presence of CHG methylation (the activity of CMT3) is not necessarily connected with H3K9me2. This situation has already been documented in tobacco by ChIP, where high CHG methylation in transcriptionally silenced *P35S* was unexpectedly accompanied by H3K9 acetylation and H3K4me3 marks [61]. H3K4me3 activation marks coexisted with methylated cytosines also in many human promoters, whose activity was frequently unaffected by the introduction of methylation marks [62].

In our system, the levels of GFP silencing strongly correlated with *P35S* methylation (Fig. 2e), indicating that promoter activity could be smoothly modulated by gradually increasing methylation levels. Recently, it was demonstrated that the effect of DNA methylation in P35S depended on the position of methylcytosines, including strand affiliation [63]. In our case, methylation was induced on long P35S region, so there was no step change in the GFP transcription connected with methylation of specific cytosine or passing over a hypothetical threshold methylation level, but instead, an even regulation was possible along with the wide range of methylation levels.

### Methylation was induced even on DNA free of methylcytosines

It has long been known that the presence of sRNAs can effectively trigger de novo RdDM of euchromatic *loci*. However, Pol V, which is considered to be involved in the final step of all canonical and non-canonical RdDM pathways [3, 43], should be attracted exclusively to methylated DNA through its interaction with SRA-domain proteins SUVH2/9 [40, 64]. Since DNA methylation is not commonly present in euchromatic DNA, the precise mechanism of de novo methylation of epigenetically naive *loci* remains unclear. In our experiments, the target and adjacent promoter regions were practically free of methylated cytosines just before treatment in two out of the three tested lines (lines 8 and 35), but in both these lines, the expression of *P35S* hairpin triggered de novo cytosine methylation. Moreover, the progression and speed of de novo methylation in line 8 were fully comparable with line 19 characterised by a relatively high level of initial CG methylation. Since methylated CG should be specifically recognised by SUVH2 [64], the impact of pre-existing cytosine methylation for Pol V activity or the role of Pol V in de novo DNA methylation remains disputable.

The independence of Pol V activity from the pre-existing methylation was recently documented in *suvh2/9* mutants, which were not significantly impaired in de novo methylation of *LTR* (long-terminal repeat) from an exogenous TE introduced into the *Arabidopsis* genome [43]. Whereas SUVH2/9 might be omitted, the authors showed that Pol V was indispensable for methylation of newly introduced TEs in T1 plants. On the contrary, it was recently shown that Pol V was not needed for the methylation of viral DNA [65], indicating the existence of an alternative pathway responsible for the establishment of DNA methylation, at least on certain occasions. In invertebrates and yeasts, which lack specialised RdDM polymerases, Pol II is supposed to serve as an enzyme assisting in the targeting of RdDM or RNA-directed histone modifications [40]. Therefore, based on the current knowledge, the involvement of Pol II in the initial targeting of de novo methylation cannot be excluded even in plants, whereas Pol V remains indispensable for a specific recognition of TE (LTR) sequences and later efficient maintenance RdDM via the canonical pathway.

## Conclusions

Methylation of cytosines in the promoter region is known to down-regulate expression of a downstream gene. In our study, we analysed the timing of transcriptional silencing of the *GFP* gene in proliferating tobacco BY-2 cell lines, which provided a highly synchronised and homogeneous response. This model enabled us to demonstrate, to our knowledge for the first time, that the induction of DNA methylation and subsequent transcriptional silencing can be a rapid process, initiated practically immediately with the appearance of promoter-specific siRNAs. These siRNAs were mostly 21- and 22-nt long and gradually accumulated at very high levels, forming up to 2.5% of all detected siRNAs on day 10. Relative distribution of siRNAs strongly differed along the promoter sequence with no transitivity observed in the target region. Our data also indicated that CG methylation occurring in the target region before the treatment did not affect the speed of RdDM. The dynamics of DNA methylation differed depending on the cytosine sequence context with gradually increasing methylation in the symmetrical CG and CHG contexts during the whole 10-day treatment, while CHH methylation reached its maxima already after 2 days and then slightly decreased. During the 2-day exposure to siRNAs, which was sufficient for the establishment of dense methylation in the target region, a gradual increase in the proportion of methylated cytosines smoothly attenuated promoter activity.

## Methods

### Plant materials

The *Nicotiana tabacum L.* cell line BY-2 [48] was cultivated in a medium based on the Murashige and Skoog (MS) [66] formula; MS salts (Merck) were supplemented with 200 mg/L K_2_HPO_4_, 100 mg/L myo-Inositol, 3% sucrose, 1 mg/L vitamin B1, and 1 µM 2,4-d, pH adjusted to 5.8 with 1 M KOH. Cell cultures were cultivated at 27 °C in darkness in 100 mL Erlenmeyer flasks on an orbital shaker at 110 rpm. The cell lines were subcultured weekly by 0.7 mL into 30 mL fresh media, and continually exponential cultures were subcultured every 3–4 days by 1.5 mL. Non-homogeneous cultures (in respect of GFP expression) were subcloned [50] before starting the experiments. A BY-2 line carrying smRS-GFP (called simply GFP in the paper) [67] stably expressed under the control of constitutive *CaMV 35S* promoter (*P35S*) for many years (Fig. 1a) [50]. The line was super-transformed with a hairpin construct prepared from PCR amplified 379-bp-long segments of the *P35S* arranged as a head-to-head inverted repeat, separated by an intron originating from the *PsbO1* gene of *Solanum tuberosum* (*PUT*-*157a*-*Solanum_tuberosum*-*62673150*) [68] with short adjacent regions (Fig. 1a) [69]. Expression of the hairpin was controlled by the *β*-estradiol (Sigma) *XVE*-inducible system [70]. Selected super-transformed lines were treated by adding *β*-estradiol to a final concentration of 2 μM (from 20 mM stock solution in DMSO stored at − 20 °C) into the cultivation media; controls were treated with a corresponding concentration of DMSO.

### Fluorescence analysis

For fluorescence analysis, protoplasts were prepared by taking 1.5 mL of the cell culture into a 2 mL tube, and the medium was drained off with cellulose wadding tampons. 1.5 mL of a protoplast enzyme mixture (10 g/L Cellulase, 1 g/L Pectolyase Y-23 in 0.45 M d-mannitol) was added, and the whole mixture was transferred into a 6-well cell-culture plate and incubated in the dark for 3 h at 26 °C with shaking 90 rpm on an orbital shaker. Protoplasts were sedimented in a 2 mL tube at 200 RCF for 5 min, and the pellet was resuspended with 1 mL of MS with 0.4 M sucrose. Protoplasts were floated by centrifugation (200 RCF for 5 min) without braking. 200 µL of the upper phase was used for flow-cytometry analysis using BD LSR II. Measured particles were first gated to select live protoplasts (Additional file 1A) and analysed using FlowJo vX.0.7 (https://www.flowjo.com/).

### Transcription analysis

Transcript levels of the *GFP* and the *P35S* hairpin were analysed by RT qPCR. RNA was isolated from 100 mg of biomass using the RNeasy^®^ Plant Mini Kit (QIAGEN) and reverse transcribed with RevertAid Reverse Transcriptase (Thermo Fisher Scientific) using anchored oligoT_23_ primer. Quantification of the *GFP* and the *P35S* hairpin transcript levels was performed on a LightCycler 480 (Roche) using the iQ TM SYBR Green Supermix (BioRad, Hercules, USA) with primers, as listed in Additional file 8: Table S1. All reactions were performed in triplicate. The PCR product specificity was verified by melting curve analysis using a LightCycler 480 software. The PCR efficiency and Cq values were calculated using the software LinRegPCR 2017.1 [71]. Calculated concentrations were normalised to the expression of the internal expression standard *EF1α* [72] with primers adopted for tobacco *EF1α* genes [73].

### DNA-methylation analysis

DNA-methylation analysis was performed using bisulfite conversion with the EpiTect Bisulfite Kit (QIAGEN) as described previously [74]. Primers for amplification of *P35S* region (Additional file 8: Table S1) were designed to anneal on the converted DNA, irrespective of its original methylation state. About ten cloned PCR products for each sample were sequenced and analysed using the MS Excel 2016 and Python 3. The level of methylation was further confirmed by qPCR after cleavage of genomic DNA with McrBC endonuclease (New England Biolabs) specific for methylated DNA (modified from [75]). In brief, 100 ng of DNA was first fragmented with a restriction enzyme that does not cut it the region of interest (AseI), and then, the reaction was split into half and supplemented with 10 units of McrBC enzyme or equivalent amount of 50% (v/v) glycerol. The DNA was digested for 6 h at 37 °C, and then, the enzyme was inactivated (20 min, 65 °C). 1 ng was used for qPCR performed as described in the section “Transcription analysis” with primers listed in Additional file 8: Table S1.

### Small RNA analysis

The RNA samples were isolated from 100 mg (FW) of BY-2 cells with the RNeasy Plant Mini Kit (QIAGEN). RNA was quality assessed and quantified. A fraction of sRNAs ranging in size from 18 to 45 nt were excised and recovered from 15% urea–polyacrylamide gels. Extracted sRNAs were ligated with 5′ and 3′ RNA adapters with T4 RNA ligase. The adapter-ligated small RNAs were subsequently transcribed into cDNA by Super-Script II Reverse Transcriptase (Invitrogen) and amplified using adaptor-specific primers. The amplified cDNA products were size-purified and circularised (ssDNA circles). This sRNA library was sequenced using the combinatorial probe–anchor synthesis (cPAS)-based BGISEQ-500 sequencer provided at an affordable price (BGI, Shenzhen, China), which was previously shown to provide highly reproducible results comparable with other NGS platforms [76]. Obtained raw data were analysed in the software Geneious 11.1.5 (https://www.geneious.com) and MS Excel 2016; only perfectly matching siRNAs were used for analyses. The sRNA data sets used in this study are available in the following database European Nucleotide Archive PRJEB32154 (http://www.ebi.ac.uk/ena/data/view/PRJEB32154).

## Supplementary information

**Additional file 1.** Analysis of GFP fluorescence in BY-2 Protoplasts.

**Additional file 2.** Distribution of methylated cytosines in the P35S region.

**Additional file 3.** Estimation of the P35S methylation by McrBC cleavage.

**Additional file 4.** Quality scores for sRNA sequencing.

**Additional file 5.** Distribution of siRNAs along the two T-DNAs before and after 10-day treatment with β-estradiol.

**Additional file 6: Table S2.** Characterization of sequenced siRNAs (siRNA numbers per 1 million reads).

**Additional file 7: Table S3.** List of the most frequent siRNAs matching with the *P35S* target (numbers of detected siRNAs per 1 million reads).

**Additional file 8: Table S1.** List of primers used in the study.

## Data Availability

The sRNA data sets used in this study are available in the following database European Nucleotide Archive PRJEB32154 (http://www.ebi.ac.uk/ena/data/view/PRJEB32154).
