# Supplementary material for: Detailed insight into the dynamics of the initial phases of de novo RNA-directed DNA methylation in plant cells
Source: Epigenetics Chromatin. 2019 Sep 11;12:54. doi: 10.1186/s13072-019-0299-0 (PMC6737654; doi:10.1186/s13072-019-0299-0)
Supplement: Supplementary file 1 — Additional file 1. Analysis of GFP fluorescence in BY-2 Protoplasts. [file 13072_2019_299_MOESM1_ESM.pdf]

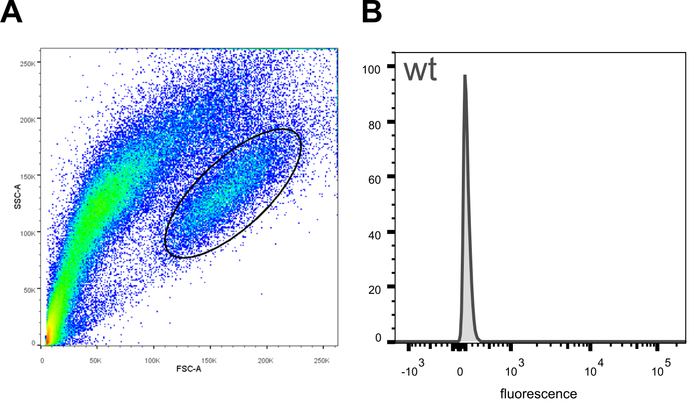

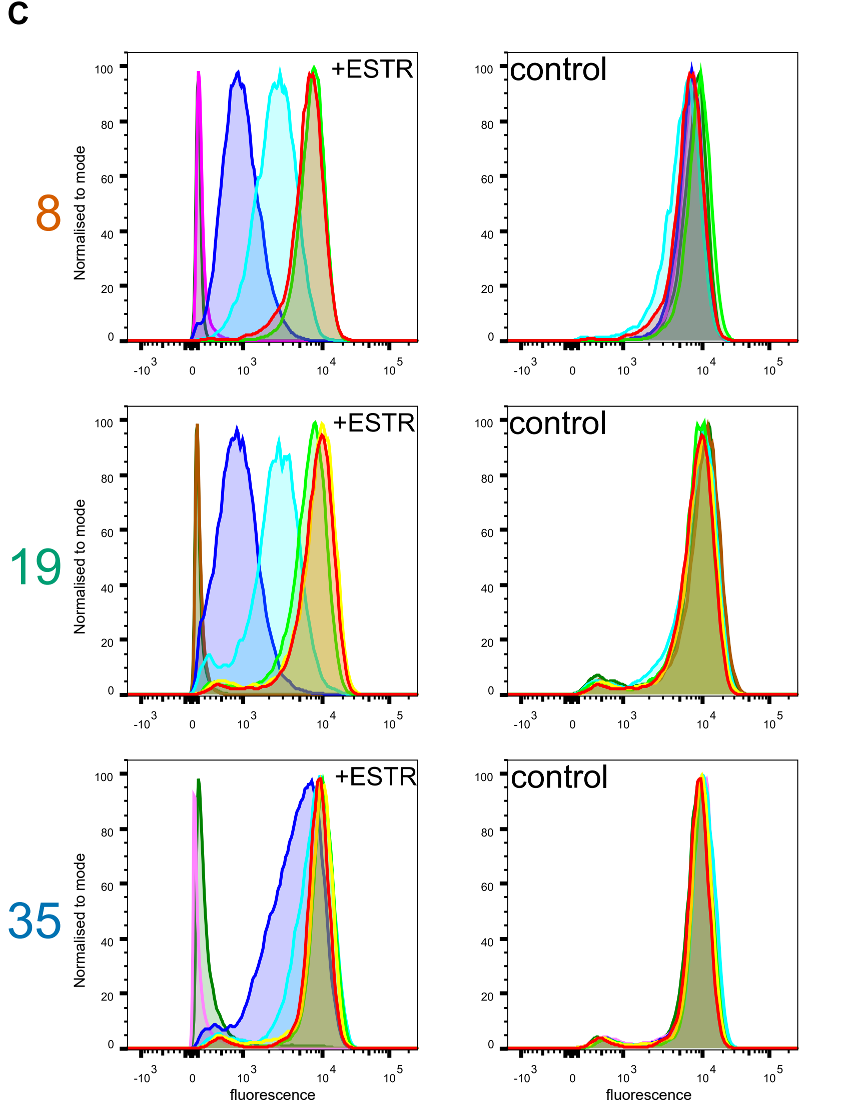

D

line		time [hours]	fluorescence (+ESTR)	fluorescence (control)
8		0	7432	7064
		24	8114	9577
		48	3023	5951
		72	1153	7414
		168	175	7612
		240	122	8505
19		0	9669	9669
		12	9987	10238
		24	7666	9601
		48	3187	9946
		72	1099	xxx
		192	154	11688
		240	144	10662
35		0	9397	9397
		12	10028	10011
		24	10846	10471
		48	8967	11179
		72	5578	9806
		168	275	10509
		240	580	9192
wt				129

Additional file 1 Analysis of GFP fluorescence in BY-2 protoplasts.

Changes in GFP fluorescence in protoplasts isolated from BY-2 suspension cultures were monitored by flow cytometry: (A) a selection of living protoplasts was based on the forward and side scatter signals; Flow-cytometry histograms show distribution of fluorescence intensity in (B) wt BY-2 cells and (C) analysed transgenic lines (8, 19 and 35) before and during the treatment with β-estradiol (+ESTR) or DMSO (control); (D) the colour code for the histograms and the table of mean GFP fluorescence intensities during the treatment.
