# Supplementary material for: Detailed insight into the dynamics of the initial phases of de novo RNA-directed DNA methylation in plant cells
Source: Epigenetics Chromatin. 2019 Sep 11;12:54. doi: 10.1186/s13072-019-0299-0 (PMC6737654; doi:10.1186/s13072-019-0299-0)
Supplement: Supplementary file 2 — Additional file 2. Distribution of methylated cytosines in the P35S region. [file 13072_2019_299_MOESM2_ESM.pdf]

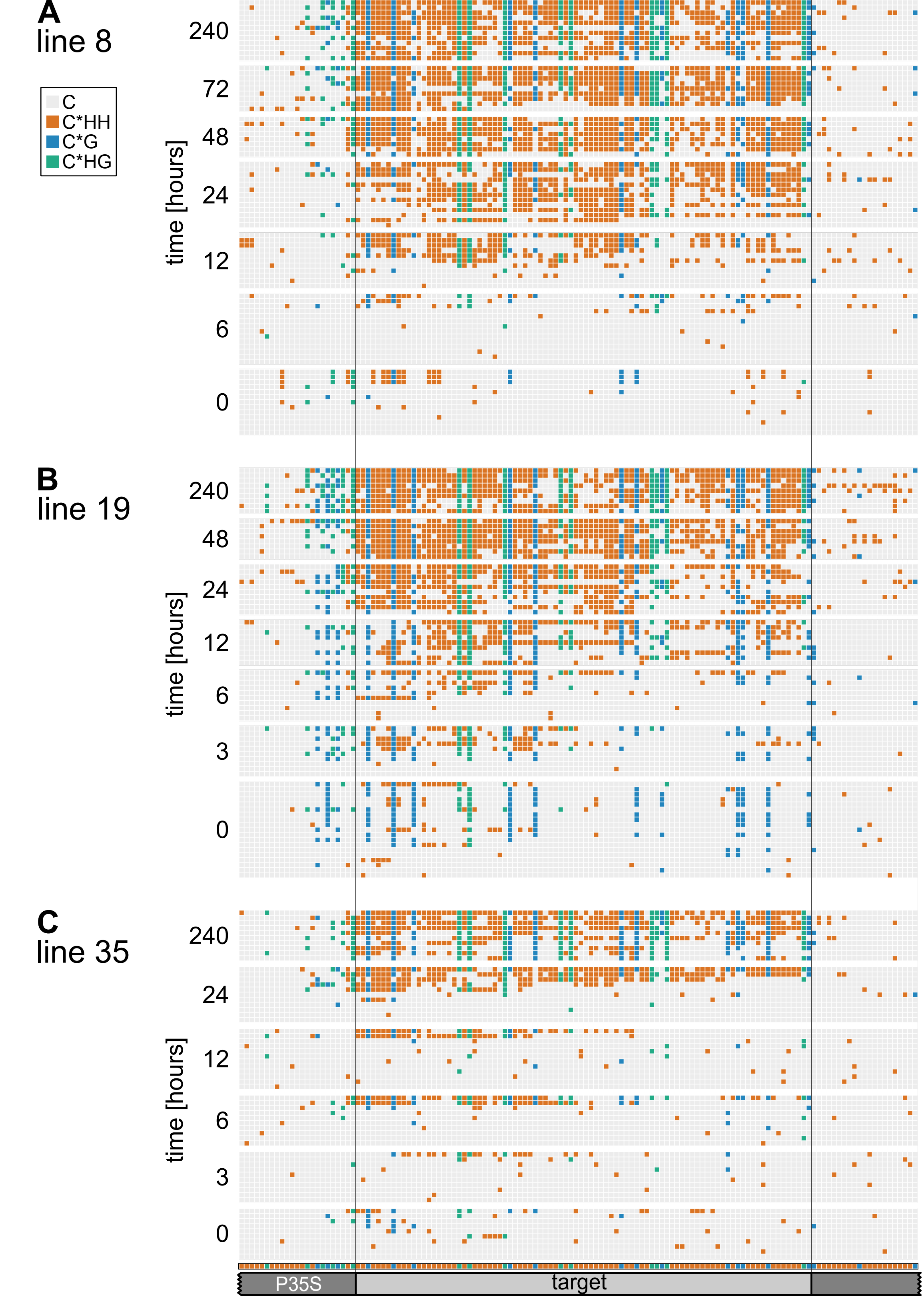

Additional file 2 Distribution of methylated cytosines in the P35S region.
Dot plot overview of the fate of all analysed cytosines in each individually sequenced read during the β-estradiol treatment of the three analysed lines. Colour-coded spots depict the position of methylated cytosines in various sequence contexts. The lowest line indicates cytosine sequence contexts in the individual positions.
