# Supplementary material for: Detailed insight into the dynamics of the initial phases of de novo RNA-directed DNA methylation in plant cells
Source: Epigenetics Chromatin. 2019 Sep 11;12:54. doi: 10.1186/s13072-019-0299-0 (PMC6737654; doi:10.1186/s13072-019-0299-0)
Supplement: Supplementary file 3 — Additional file 3. Estimation of the P35S methylation by McrBC cleavage. [file 13072_2019_299_MOESM3_ESM.pdf]

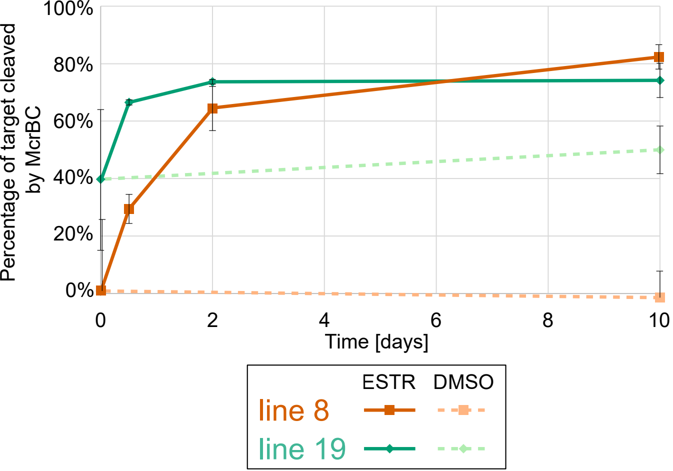

Additional file 3 Estimation of the P35S methylation by McrBC cleavage.
The plot indicates the proportion of methylated DNA at selected time points, as determined by qPCR of the P35S region after treatment with methyl-specific activity of McrBC.
