# Supplementary material for: Detailed insight into the dynamics of the initial phases of de novo RNA-directed DNA methylation in plant cells
Source: Epigenetics Chromatin. 2019 Sep 11;12:54. doi: 10.1186/s13072-019-0299-0 (PMC6737654; doi:10.1186/s13072-019-0299-0)
Supplement: Supplementary file 4 — Additional file 4. Quality scores for sRNA sequencing. [file 13072_2019_299_MOESM4_ESM.pdf]

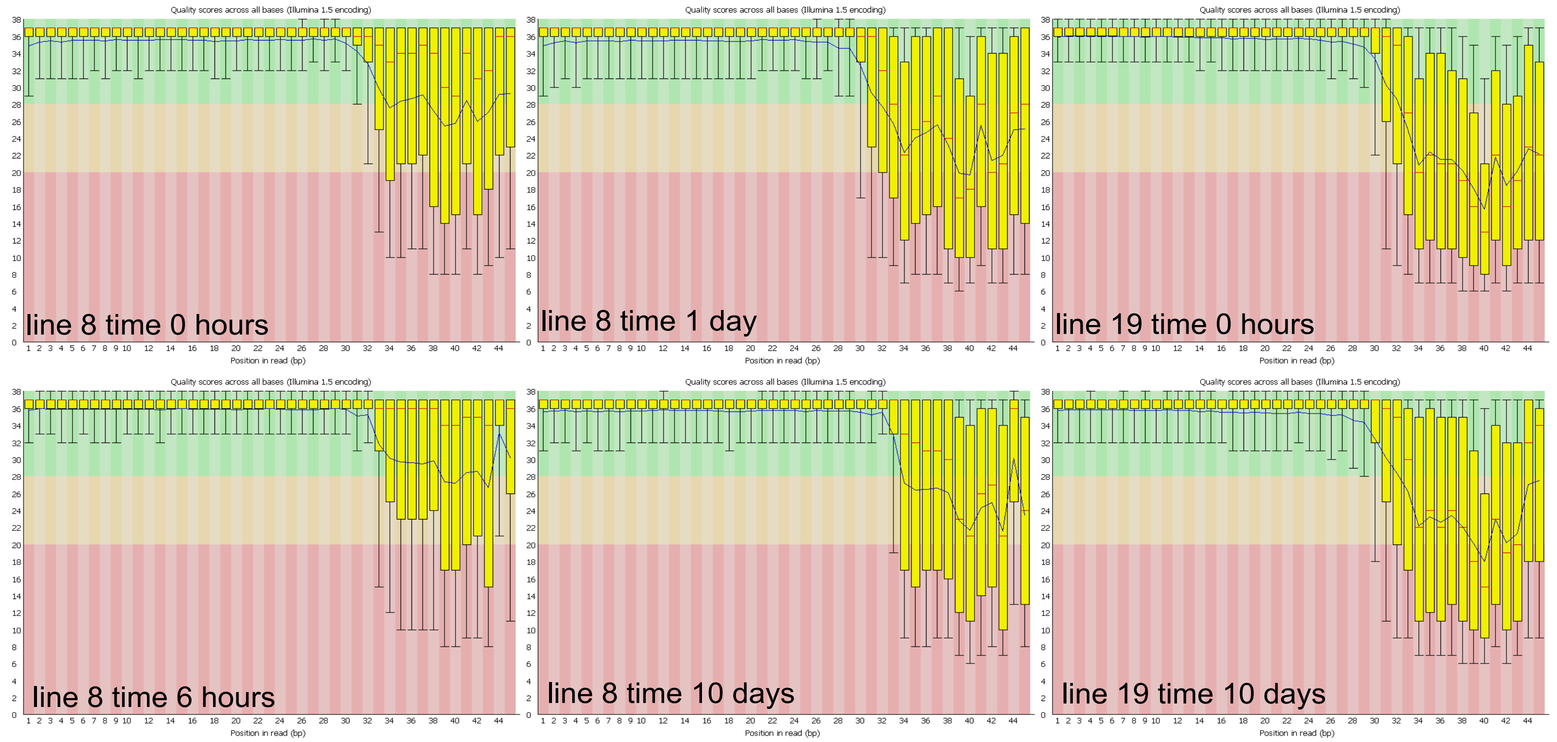

Additional file 4 Quality scores for sRNA sequencing.

The plots of “Illumina 1.5 encoding” quality scores clearly show high reliability of sequencing of sRNAs up to the length of about 30nt.
