# Supplementary material for: Detailed insight into the dynamics of the initial phases of de novo RNA-directed DNA methylation in plant cells
Source: Epigenetics Chromatin. 2019 Sep 11;12:54. doi: 10.1186/s13072-019-0299-0 (PMC6737654; doi:10.1186/s13072-019-0299-0)
Supplement: Supplementary file 5 — Additional file 5. Distribution of siRNAs along the two T-DNAs before and after 10-day treatment with β-estradiol. [file 13072_2019_299_MOESM5_ESM.pdf]

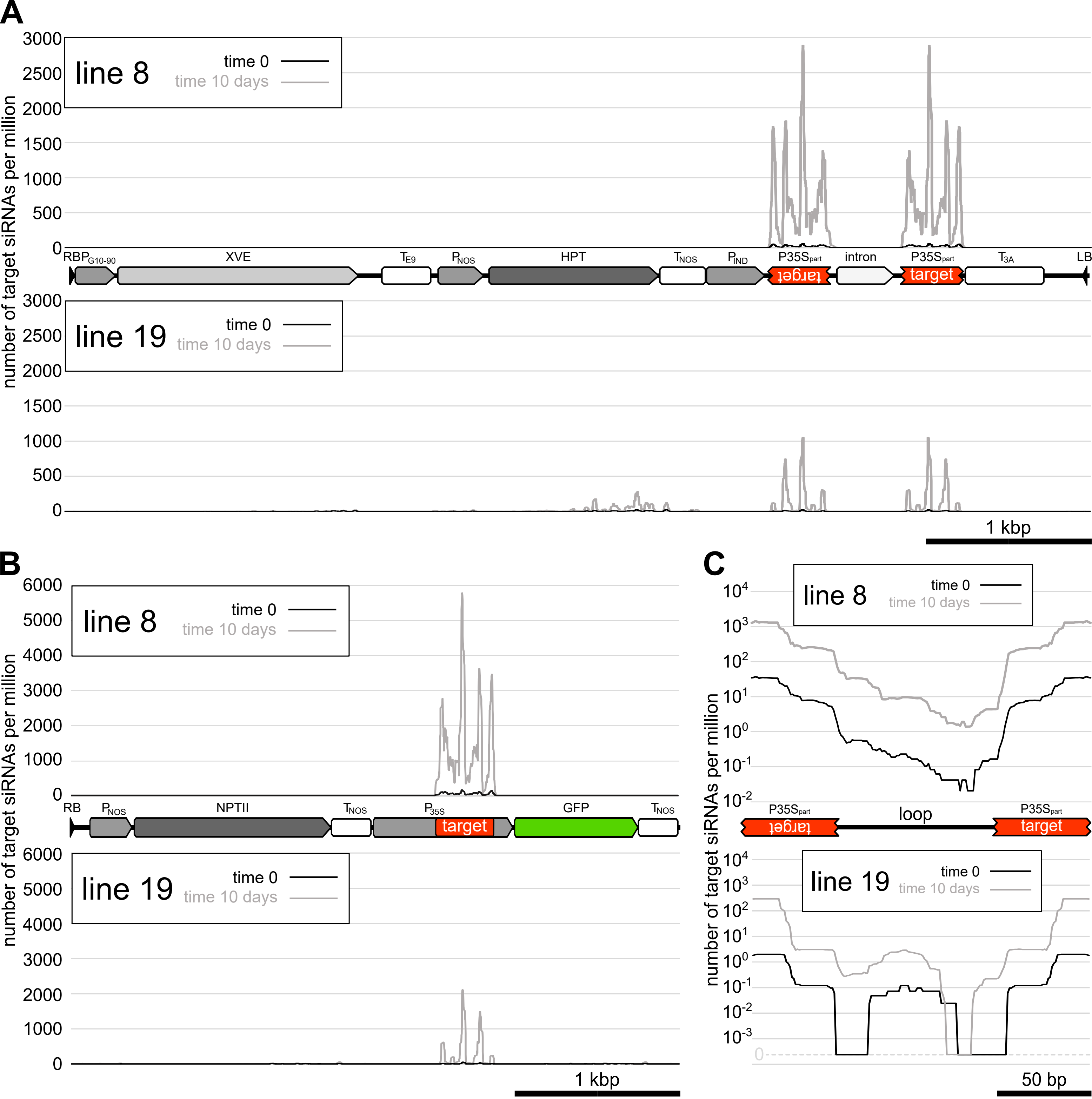

Additional file 5 Distribution of siRNAs along the two T-DNAs before and after 10-day treatment with β-estradiol.

siRNAs matching with (A) the inducer (unspliced) and (B) the target T-DNAs are depicted as the numbers per million of all sequenced siRNAs (note different scales for the two lines); (C) close-up of the distribution of siRNAs along the spliced spacer region of the hairpin transcript (in a logarithmic scale).
