# Supplementary material for: Detailed insight into the dynamics of the initial phases of de novo RNA-directed DNA methylation in plant cells
Source: Epigenetics Chromatin. 2019 Sep 11;12:54. doi: 10.1186/s13072-019-0299-0 (PMC6737654; doi:10.1186/s13072-019-0299-0)
Supplement: Supplementary file 6 — Additional file 6: Table S2. Characterization of sequenced siRNAs (siRNA numbers per 1 million reads). [file 13072_2019_299_MOESM6_ESM.docx]

**Table S2** Characterization of sequenced *siRNAs* (*siRNA* numbers per 1 million reads)

| **siRNA target** | **line 8** | | | | **line 19** | |
| --- | --- | --- | --- | --- | --- | --- |
|  | **0** | **6 hours** | **1 day** | **10 days** | **0** | **10 days** |
| silencer T-DNA (6 150 bp long) | 702 | 4 051 | 15 645 | 24 549 | 373 | 6 963 |
| target T-DNA (5 300 bp long) | 698 | 4 039 | 15 604 | 24 455 | 363 | 5 788 |
| *P35S* target (379 bp long) | 693 | 4 033 | 15 596 | 24 448 | 127 | 5 165 |
| *Tnt1* TE (363 bp long) | 70 | n.d. | n.d. | 61 | 20 | 14 |
| *LTR* of *Tto1* TE (574 bp long) | 1 235 | n.d. | n.d. | 840 | 613 | 414 |
| ***P35S* siRNA size** |  | | | | | |
| <20 nt | 7 | 29 | 141 | 369 | 1 | 50 |
| 20 nt | 22 | 163 | 499 | 997 | 3 | 128 |
| 21 nt | 447 | 2 430 | 10 068 | 16 640 | 67 | 3 107 |
| 22 nt | 181 | 1 189 | 4 054 | 5 348 | 44 | 1 562 |
| 23 nt | 12 | 88 | 260 | 338 | 3 | 90 |
| 24 nt | 23 | 126 | 553 | 737 | 9 | 225 |
| 25 nt | 0 | 4 | 14 | 14 | 0 | 3 |
| >25 nt | 0 | 4 | 8 | 5 | 0 | 0 |
| **total number of sequenced siRNAs** | 50 305 789 | 45 380 666 | 49 032 131 | 49 421 790 | 41 774 120 | 40 590 644 |
