# Supplementary material for: Detailed insight into the dynamics of the initial phases of de novo RNA-directed DNA methylation in plant cells
Source: Epigenetics Chromatin. 2019 Sep 11;12:54. doi: 10.1186/s13072-019-0299-0 (PMC6737654; doi:10.1186/s13072-019-0299-0)
Supplement: Supplementary file 7 — Additional file 7: Table S3. List of the most frequent siRNAs matching with the P35S target (numbers of detected siRNAs per 1 million reads). [file 13072_2019_299_MOESM7_ESM.docx]

**Table S3.** List of the most frequent siRNAs matching with the *P35S* target (numbers of detected siRNAs per 1 million reads)

| **line 8** | | | | | | |
| --- | --- | --- | --- | --- | --- | --- |
|  |  |  | **time** | | | |
| **sequence** | **orientation** | **length** | **0** | **6 hours** | **1 day** | **10 days** |
| TTGCCCAGCTATCTGTCACTT | forward | 21 nt | 24 | 155 | 539 | 1378 |
| TCGTGCTCCACCATGTTGACG | reverse | 21 nt | 18 | 75 | 422 | 747 |
| TTCCAACCACGTCTTCAAAGC | forward | 21 nt | 18 | 126 | 390 | 726 |
| TTTGGGACCACTGTCGGCAGA | reverse | 21 nt | 13 | 46 | 287 | 779 |
| TCCTCGGATTCCATTGCCCAG | forward | 21 nt | 8 | 32 | 200 | 798 |
| TCTTCAAAGCAAGTGGATTGA | forward | 21 nt | 14 | 46 | 314 | 521 |
| CATTGCCCAGCTATCTGTCACT | forward | 22 nt | 9 | 67 | 210 | 487 |
| TTTTTGGAGTAGACAAGTGTG | reverse | 21 nt | 14 | 62 | 383 | 293 |
| TTTCCGGATATTACCCTTTGT | reverse | 21 nt | 8 | 36 | 165 | 477 |
| CAACCACGTCTTCAAAGCAAG | forward | 21 nt | 6 | 32 | 141 | 407 |
| ATGGCCTTTCCTTTATCGCAA | reverse | 21 nt | 10 | 24 | 237 | 242 |
| TTGAAGATGCCTCTGCCGACA | forward | 21 nt | 9 | 56 | 180 | 206 |
| TTGCCCAGCTATCTGTCACTTT | forward | 22 nt | 5 | 37 | 125 | 277 |
| ATTGCCCAGCTATCTGTCACT | forward | 21 nt | 7 | 45 | 151 | 237 |
| TGCTCCACCATGTTGACGAAG | reverse | 21 nt | 4 | 21 | 80 | 307 |
|  | | | | | | |
| **line 19** | | | | | | |
|  |  |  | **time** | | | |
| **sequence** | **orientation** | **length** | **0** |  |  | **10 days** |
| TTGCCCAGCTATCTGTCACTT | forward | 21 nt | 10 |  |  | 461 |
| TCGTGCTCCACCATGTTGACG | reverse | 21 nt | 8 |  |  | 334 |
| TTTGGGACCACTGTCGGCAGA | reverse | 21 nt | 5 |  |  | 292 |
| CATTGCCCAGCTATCTGTCACT | forward | 22 nt | 7 |  |  | 281 |
| TCCTCGGATTCCATTGCCCAG | forward | 21 nt | 4 |  |  | 207 |
| TTGCCCAGCTATCTGTCACTTT | forward | 22 nt | 5 |  |  | 155 |
| TTGAAGATGCCTCTGCCGACA | forward | 21 nt | 2 |  |  | 137 |
| ATTGCCCAGCTATCTGTCACTT | forward | 22 nt | 4 |  |  | 129 |
| TTGAAGATGCCTCTGCCGACAG | forward | 22 nt | 3 |  |  | 119 |
| TCGTTGAAGATGCCTCTGCCG | forward | 21 nt | 2 |  |  | 115 |
| TGCTCCACCATGTTGACGAAG | reverse | 21 nt | 2 |  |  | 111 |
| TTGGGACCACTGTCGGCAGAG | reverse | 21 nt | 2 |  |  | 95 |
| CTCCTCGGATTCCATTGCCCAG | forward | 22 nt | 2 |  |  | 90 |
| CTTTGGGACCACTGTCGGCAG | reverse | 21 nt | 2 |  |  | 91 |
| ATTGCCCAGCTATCTGTCACT | forward | 21 nt | 2 |  |  | 82 |
