# Supplementary material for: Detailed insight into the dynamics of the initial phases of de novo RNA-directed DNA methylation in plant cells
Source: Epigenetics Chromatin. 2019 Sep 11;12:54. doi: 10.1186/s13072-019-0299-0 (PMC6737654; doi:10.1186/s13072-019-0299-0)
Supplement: Supplementary file 8 — Additional file 8: Table S1. List of primers used in the study. [file 13072_2019_299_MOESM8_ESM.docx]

**Table S1** List of primers used in the study

| **Primer** | **Sequence (5‘ -> 3‘)** | **Used for** |
| --- | --- | --- |
| M13F | GTAAAACGACGGCCAGT | sequencing |
| M13R | AACAGCTATGACCATG |  |
| BS_35S_FG1 | GYAAGTAATAGAGATTGGAG | PCR from DNA after bisulfite modification |
| BS_35S_RC2 | AAAARTTCTTCTCCTTTACTC |  |
| 35S_Nuc1_F | AACAGAACTCGCCGTAAAGAC | DNA methylation by McrBC |
| 35S_Inter1_R | GTCTCAATTGCCCTTTGGTCT |  |
| EF1α_F | TGAGATGCACCACGAAGCTC | qPCR |
| EF1α_R | CCAACATTGTCACCAGGAAGTG |  |
| GFP3_F | GAGACACCCTCGTCAACAGG |  |
| GFP3_R | TGGTCTGCTAGTTGAACGCTT |  |
| MSPIII_F | AACCATGCCTTACAATCAC |  |
| MSPIII_R | GACACAAAGCATAAAGCAG |  |
